# Documentation and dissemination of scientific evidence by the Uganda Public Health Fellowship Program: experiences and lessons learnt, 2015–2020

**DOI:** 10.1186/s12960-021-00665-1

**Published:** 2021-10-21

**Authors:** Lilian Bulage, Alex Riolexus Ario, Steven N. Kabwama, Benon Kwesiga, Daniel Kadobera, Christine Kihembo, Simon Antara, Rhoda K. Wanyenze

**Affiliations:** 1 Uganda Public Health Fellowship Program, Kampala, Uganda; 2 African Field Epidemiology Network, Kampala, Uganda; 3 Ministry of Health, Kampala, Uganda; 4 Makerere University School of Public Health, Kampala, Uganda

**Keywords:** Generated scientific evidence, Field Epidemiology Training Programs, Documentation, Dissemination, Research–practice gap, Uganda

## Abstract

**Background:**

During participation in Field Epidemiology Training Programs (FETP) residents/fellows generate scientific evidence from the various public health projects they are engaged in. However, this evidence is not sufficiently disseminated to influence policy and practice. We describe the processes through which evidence is disseminated, and share achievements and lessons learnt during the first 5 years of the Uganda Public Health Fellowship Program (PHFP).

**Methods:**

The PHFP is a 2-year, full-time, non-degree fellowship, and the first post-masters FETP in Africa for mid-career public health professionals. Fellows gain competencies in seven main domains, which are demonstrated by deliverables while learning through service delivery, 80% of the time within Ministry of Health and related agencies. Generated public health evidence is disseminated immediately through sharing of daily situation reports with the National Task Force for Epidemic Preparedness and Response, as well as regional and district levels. Information is also disseminated on an intermediate to long-term basis through newspaper articles, epidemiological bulletins, abstracts and conference presentations, and publications in scientific journals.

**Results:**

During 2015–2020, PHFP enrolled 80 fellows in seven cohorts, including five of whom who had graduated. Overall, 355 field projects had been implemented. Additionally, PHFP made 287 conference presentations including 108 international and 178 national conferences. Altogether, the Uganda PHFP has received 7 awards, 4 of these for excellent scientific presentations during conferences. By end of 2020, PHFP had written 147 manuscripts at different stages of peer review, including 53 publications; and published 153 epidemiological bulletins. Dissemination performance was limited by delays due to challenges like non-adherence to product clearance guidelines, limited persons to conduct product review, and limited expertise on certain scientific areas, authorship related issues, and competing priorities among fellows, staff, and alumni.

**Conclusions:**

The PHFP has disseminated public health evidences through various means to a wider range of audiences within Uganda and globally. Manuscript publication and monitoring of actions taken as a result of evidence dissemination is still limited. We recommend putting in place mechanisms to facilitate publication of all scientific evidence and deliberate efforts to ensure and monitor scientific evidence utilization.

## Background

Field Epidemiology Training Programs (FETP) in Africa were set up with the aim of contributing to building competencies to address shortage of human resource needs in the region. Overtime, the number of FETPs in Africa has increased from 4 in 1994 to 45 in 2018. During the course of training, FETP residents/fellows generate public health evidence from the various public health projects they implement. However, much of this evidence is not sufficiently disseminated to influence policy and practice. Dissemination of generated public health evidences is an ethical obligation and a good practice. Additionally, dissemination is essential to improve health, adoption of evidence-based interventions, and improved practice and learning across various settings [1].

A research–practice gap exists across all fields of public health and medical practice in resource-limited settings [2]. The gap between discovery of public health knowledge and application in practice and policy is due in part to ineffective knowledge translation (KT) and utilization approaches following dissemination. Knowledge translation is defined as a dynamic and iterative process that includes synthesis, dissemination, exchange and ethically sound application of knowledge to yield beneficial outcomes for society [3]. Effective KT translation requires researchers to play an active role in promoting evidence uptake. Ineffective dissemination and knowledge translation may be due to weak health systems including inadequate funding, limited human personnel, and poor infrastructure in resource-limited settings [4]. And despite many researchers and funding agencies valuing dissemination of public health evidence and eventual uptake, the practice is not routinely embraced by many FETP residents, fellows, and other health professionals in public health practice. Scientific evidence dissemination in most of the FETPs is conducted through traditional methods such as report writing and dissemination; dissertation defense and storage in libraries. Such traditional methods of scientific evidence dissemination are characterized with limited knowledge translation.

Not embracing dissemination and KT of public health evidence by FETPs may likely be due to many factors including lack of specific guidance on how best to accomplish effective dissemination and knowledge translation [5]. Ineffective KT is associated with a number of individual and institutional levels barriers, including inadequate KT knowledge and skills, particularly for communicating research and interacting with research end-users, insufficient funding, and inadequate institutional guidelines, structures and incentives promoting KT practice [6]. We describe the processes through which generated public health evidence is disseminated, share experiences and achievements, and lessons learnt during the first 5 years of existence of the Uganda Public Health Fellowship Program (PHFP).

## Methods

### Program description, 2015–2020

The PHFP is a 2-year, non-degree-granting, full-time, competency-based fellowship program modeled after the US Epidemic Intelligence Service (EIS) program. The program is the first post-masters FETP in Africa for mid-career public health professionals. Fellows gain competencies in seven main domains which are demonstrated by deliverables while learning through service delivery, 80% of the time within Ministry of Health (MoH) and other health agencies. The domains include response to a public health emergency (outbreak investigation or a rapid health assessment of displaced persons), conducting an applied epidemiologic study (HIV/AIDS, malaria, vaccination survey project, etc.), conducting public health surveillance (analysis of surveillance data and evaluation of a surveillance system), public health programing (economic evaluation and quality improvement project), and management and leadership (leading a public health initiative). Each fellow is also required to conduct an HIV/AIDS-related project. Besides the highlighted deliverables, fellows participate in Tuberculosis Operations Research Projects. Fellows generate public health evidence at the end of each of the executed deliverables. A more detailed description of the program has previously been described [7].

### Public health evidence dissemination process, 2015–2020

#### Immediate public health evidence dissemination mechanisms

##### Public health emergency response activities

The fellows and their supervisors are part of the National Rapid Response Team (NRRT). Every time they participate in public health emergency activities, daily situation reports are prepared and shared with the Public Health Emergency Operation Center (PHEOC) which eventually shares with the National Task Force (NTF) for Epidemic Preparedness and Response. The NTF, a structure under the Ministry of Health (MoH) created to coordinate emergency public health response and is chaired by the Director General of Health Services (DGHS) and co-chaired by the Director Animal Resources in the Ministry of Agriculture, Animal Industry and Fisheries, is responsible for coordination of government, development partners, and other relevant stakeholders who are involved in the response activities. The NTF is composed of a multidisciplinary team of Subject Matter Experts (SMEs) in technical areas that guide the country in preparedness and response [8]. While in the field, a preliminary report is also prepared and shared with the NTF and the local authorities where the emergency has occurred. Additionally, at the end of the investigation and response, an activity report is prepared including a power point presentation and shared with the NTF and the local authorities.

##### Applied epidemiologic studies and public health surveillance

Activity reports including power point presentations are prepared and shared with the relevant authorities including Local Governments, partners, MoH, and other line ministries.

#### Long-term public health evidence dissemination mechanisms

##### Scientific publications: quarterly epidemiological bulletins, manuscripts, and publications

*Quarterly epidemiological bulletins* On a quarterly basis, Fellows prepare bulletins and share with MoH and the NTF for Epidemic Preparedness and Response, and other relevant stakeholders. Fellows are specifically in charge of the preparation and dissemination of the Uganda National Institute of Public Health (UNIPH) quarterly epidemiological bulletin. The UNIPH bulletin routinely features updates on the latest public health emergency occurrences in the country and highlights of other epidemiological studies conducted by the Fellows, Implementing Partners and MoH, and updates from the various units of the MoH.

Each fellow is presented with at least one opportunity to take the lead in preparing the bulletin under the oversight of the supervisors, scientific writer, and the field coordinator. The bulletin is shared with the NTF by email. Hard copies of the bulletin are also periodically printed out (during graduation, and other program organized functions) and shared with the intended users.

Jointly with the respective MoH staff, fellows have also spearheaded the initiation, preparation and production of unit specific quarterly bulletins. Fellows have specifically founded and produced the malaria quarterly bulletin at the National Malaria Control Division, the National Tuberculosis and Leprosy Program quarterly bulletin, and the non-communicable diseases quarterly bulletin.

*Manuscripts and publications* As a requirement before graduation, each fellow presents to the program evidence of having submitted at least one manuscript to a peer-reviewed scientific journal for publication consideration.

*Newspaper bulletin articles* Each fellow is required to publish at least two newspaper articles focusing on either a recent public health occurrence or any other relevant public health issue in one of the national newspapers.

##### Presentations at scientific conferences

*Annual National Field Epidemiology Conference* Because of the many public health projects and scientific evidence generated annually, the program on an annual basis organizes a National Field Epidemiology Conference (NFEC) to further allow dissemination of the public health products. During the conference each fellow presents one or more of their products to the national and international audience including agencies such as the US CDC, the United States Agency for International Development (USAID), and the World Health Organization, among others.

*Other national and international conference presentations* Besides the NFEC, fellows also further disseminate their work to either a national or international level conference before graduation.

*Graduation day dissemination* During graduation, each fellow presents a summary of all the achievements during the 2-year training period. In addition to the summary, each fellow also presents their best product to the audience for further dissemination. The Fellows’ Summary Report book is disseminated to all attendees of the conference, MoH departments and affiliated institutions, and donor agencies.

*Other project disseminations* HIV/AIDS-related project and TB OR deliverables have been additionally disseminated through power point presentations to the National TB and Leprosy Program as well as the National AIDS and Sexually Transmitted Infections Program.

### Quality control and clearance of public health evidence dissemination products

Before dissemination to the public, all public health evidence dissemination products undergo a clearance process to ensure scientific rigor. Each product is either cleared by the PHFP internally, or PHFP and US CDC. Internally, each product is first reviewed by the source project respective supervisor, followed by the Field Coordinator, the US CDC Resident Advisor, and the US in-country Associate Director for Science (ADS) and CDC. The clearance process is sometimes extended to US CDC Atlanta office if there are CDC coauthors. Additionally, some products undergo review by external subject matter experts if deemed necessary. All presentations must undergo rehearsals before disseminations to the respective audiences.

## Results

### Enrollment and field projects, Uganda Public Health Fellowship Program, 2015–2020

Since inception, PHFP has enrolled 7 cohorts of 80 fellows, five of which have graduated. By December 2020, Fellows had participated in 355 field projects (33-cohort 2015, 62-cohort 2016, 59-cohort 2017, 81-cohort 2018, 79-cohort 2019, and cohort 39-2020). Of the 355 field projects, 112 were outbreak investigations, 13 were other public health emergency investigations, 86 were surveillance data analysis and surveillance system evaluations, 109 were applied epidemiological investigations, 5 were cost analysis of outbreaks, and 30 were quality improvement projects (Table 1).

**Table 1:** Field projects participated in, scientific publications, and presentations made at scientific conference, and awards received, Uganda Public Health Fellowship Program, 2015–2019

Cohort	2015	2016	2017	2018	2019	2020	Staff	Total
Field projects participated	33	62	59	81	78	39	–	355
Outbreak investigations	18	15	27	31	17	4	–	112
Emergency investigations	1	4	7	0	1	0	–	13
Surveillance data analysis	9	16	15	10	23	13	–	86
Applied epidemiologic studies	5	22	5	29	27	21	–	109
Cost analysis of outbreaks	0	2	1	0	1	1	–	5
Quality improvement	0	3	4	11	12	0	–	30
Scientific publications	37	37	59	63	57	32	15	300
Peer-reviewed, published or accepted	18	8	14	4	0	2	7	53
Peer-reviewed, submitted, not published yet	0	3	18	26	25	14	8	94
MoH quarterly Epi Bulletin articles	19	26	27	33	32	16	–	153
Presentations made at scientific conferences	55	60	66	47	38	21		287
International conferences^a^	21	26	32	14	8	7	–	108
National conferences^b^	34	34	34	32	30	14	–	178
Awards received	0	2	2	1	1	1	–	7

^a^EAHSC: East African Health and Scientific Conference; AFENET: African Field Epidemiology Network Conference 2016 and 2018; TEPHINET: Training Programs in Epidemiology and Public Health 2017 and 2019; ICEID: International Conference Emerging Infectious Diseases; EIS: Epidemiology Intelligence Service International Night; IMED: International Meeting on Emerging Diseases and Surveillance 2016; International Conference on Typhoid and Other Invasive Salmonelloses 2017, Global Health Security Agenda Ministerial Meeting, Kampala, 2017, Multilateral Initiative on Malaria Conference, Dakar, Senegal 2018, International Workshop on HIV & Women, Seattle, USA 2019, 5th World Congress on Public Health, Epidemiology & Nutrition 2018, 3rd International Federation of Environmental Health Academic Conference, Kampala, Uganda, 2019, 3rd One Health Central and Eastern Africa Conference, 2019, 19th International Congress on Infectious Diseases, International Conference on Cancer in Africa, Kigali, 2017, International Conference on AIDS and Sexually Transmitted Infections in Africa, Abidjan, 2017, East African COVID 19 Conference 2020

^b^NFEC: National Field Epidemiology Conference 2015–2019; JASH: Joint Annual Scientific Health Conference 2015–2019; USHS: Uganda, Uganda Society for Social Scientists Conference, 2015–2019, and Non-communicable Diseases Symposium, Kampala, Uganda, 2018

### Scientific publications*—*quarterly epidemiological bulletins, manuscripts, and publications, Uganda Public Health Fellowship Program, 2015–2020

Since inception in 2015, the UPHFP Fellows have published 153 epidemiological bulletins (19-cohort 2015, 26-cohort 2016, 27-cohort 2017, 33-cohort 2018, 32-cohort 2019, 16-cohort 2020, and 1-MoH) (Table 1). To date, the fellows jointly with the program staff have written 147 manuscripts which are at different stages of peer review (Table 1). Of the 147, 53 have been published. Of the 53 published manuscripts, seven were primarily written by PHFP staff. Among the 53 published articles, one was featured in global media outlets including CNN, the New York Times, the BBC, the VOA, Newsweek, and NPR. Fifty-one percent of the 147 manuscripts originated from outbreak investigations as field type source followed by articles from surveillance data analysis projects at 19.7%, and others (Table 2). Of the 147 manuscripts, seven are case-studies written by program staff (six focused on sharing practical skills of outbreak investigations based on real life outbreaks experienced in Uganda while the other aimed at reminding Field Epidemiology Fellows/Residents on how to conduct surveillance system evaluations) (Table 2). In addition to scientific publications, fellows have so far published a total of 105 newspaper articles in different media houses.

**Table 2:** Source of manuscript and article type, Uganda Public Health Fellowship Program, 2015–2020

Source project	Frequency	Percentage
Outbreak investigation	75	51.0
Surveillance data analysis	29	19.7
HIV project	15	10.2
Program evaluation	9	6.1
HIV project	8	5.4
Epidemiologic study	5	3.4
Surveillance system evaluation	3	2.0
Economic analysis	2	1.4
Quality improvement	1	0.7
Type of paper		
Main article	139	94
Case study	7	5
Short communication	1	1

### Presentations at scientific conferences, Uganda Public Health Fellowship Program, 2015–2020

To date, the fellows have made 287 conference presentations. Of the 287, nine were made during EIS, 99 during other international conferences including AFENET and TEPHINET, and 178 during national conferences. The program received an award during three out of four times we participated in EIS conferences including the Jeffrey P. Koplan Award for Excellence in Scientific Poster Presentation and Best Presentation. The program has also received 2nd runner-up best presentations during the two AFENET conferences so far participated. Altogether, the Uganda PHFP has received 7 awards, 4 of which are as a result of excellent scientific presentation skills during conferences (Table 1).

Following execution of TB/HIV projects, the fellows disseminated the findings to the National Tuberculosis and Leprosy and the National AIDS Control Programs. These findings were further disseminated at the HIV science summit.

## Discussion

Within the first 5 years of existence, the Public Health Fellowship Program has contributed to global health security through ensuring that all scientific evidence generated during execution of public health projects is disseminated in a timely manner at all levels of the health care system nationally and globally using various means. Most of the published manuscripts originated from outbreak investigations and surveillance data analysis field products. The design of the PHFP, i.e., where fellows are hosted by priority departments within MoH Uganda for 80% of their 24-month training period allows them to become fully integrated into the ministry structure and hence easy of dissemination of all products right from grass root levels. Also, the PHFP and Fellows are part of the NTF and the NRRT, which offers timely opportunities for participation in response to public health emergencies and dissemination of information for evidence-based decision-making. The various means (daily situation reports, activity reports, newspaper articles, epidemiological bulletins, conference presentations, and manuscript publications) through which different types of generated scientific evidences can be disseminated has also contributed to PHFPs current story. The various means allows dissemination to a wider audience at the appropriate time. Additionally, PHFP being a post-masters fellowship, admits persons with some basic baseline level of scientific dissemination knowledge and skills hence individuals are already prepared to grasp the taught skills and knowledge and hence the achievements so far reached. The robust system through which the quality of the dissemination products is checked right from the field through activities such as review by the different levels of command within the PHFP, and externally by subject matter experts, and must do rehearsals before conference presentations increases scientific rigor of the generated public health evidence and the chances of dissemination at different levels. The PHFP through support from the African Field Epidemiology Network (AFENET) hired a scientific writer in the year 2017 to oversee manuscript writing among other products. The writer organizes manuscript writing meetings and offers technical assistance during manuscript write-up, and also follows up on the manuscripts at the different stages of clearance among other responsibilities. Presence of the scientific writer could be contributing to the good performance.

Most of the publications have originated from outbreak investigations followed by surveillance data analysis field projects. Most publications having originated from outbreak investigation is not surprising because fellows usually participate in such activities during the 1st year of training, which allows ample time for turning the respective reports in manuscripts and hence publication. Secondly, other field source projects such as surveillance data analysis projects are associated with missing data and values which creates delays in publication. Therefore, PHFP has taken advantage of the fact that they have almost hundred percent control over the quality of data and evidence generated during public health emergencies such as outbreak investigations. And because we generate generally good quality data, it is easy to publish work out of such field activities. Additionally, Uganda has been experiencing a number of re-emerging and emerging infectious outbreaks which presents unique and important data to share with the rest of the world and hence easy of writing and publication [9]. Notable are Crimean Congo Hemorrhagic fever, anthrax, and yellow fever among others. We observe that not many of the HIV products and quality improvement products are disseminated to manuscript level and hence publication. The limited publications out of HIV and quality improvement projects is likely because most of the fellows complete such projects towards the end of the 2nd year hence limited time for writing due to many competing priorities.

Despite the achievements so far, PHFP’s performance is being limited by the long waiting times to have all the products that must be cleared up to the funding agency level. However, over time, PHFP has identified issues such as delay in starting to put together dissemination products despite having all it takes and failure to adhere to product clearance guidelines by the fellows. Delays in dissemination have also been attributed to limited number of people dedicated to review of products, lack of expertise in certain technical areas which leads to back and forth movements during the clearance of the products, authorship level challenges, and competing priorities by the fellows and the PHFP staff, failure to disseminate, specifically to publish all publishable products post-graduation due to competing priorities. To address the highlighted challenges, PHFP has identified the best start time for each of the products and the minimum and maximum time it takes each type of product to get ready for dissemination. For instance, fellows are supposed to prepare abstracts out of field activities as soon as the reports are ready and ensure immediate submission for review and clearance. Immediate abstract preparation and submission for clearance allows ample time for clearance and eventually leads to many good quality products ready for dissemination at the appropriate time, audience, and place. We have also observed that by around September of the 1st year of training, most of the fellows have a well-done field projects that could be translated to manuscripts. Additionally, having a mix of short time theoretical lectures on scientific writing and real hands on writing based on field project reports is more fruitful as opposed to independent theoretical classes and individual writing. Because of the observations and realized benefits of different approaches, our advanced class for scientific writing includes brief theoretical lectures on scientific writing and real hands on practical writing based on field reports. Holding the scientific writing class during the 1st year of training allows ample time to ensure product quality and fulfill all the clearance recommendations. The PHFP now identifies and works with subject matter experts to ensure scientific integrity of products whenever necessary, which practice limits the back and forth movements of products over technical issues. The PHFP encourages everyone to follow the criteria for authorship as stipulated by different guidelines, but also to talk about authorship right from the start of each dissemination products.

## Limitations

Despite the dissemination efforts, we do not have evidence for use of each of disseminated information for evidence-based interventions and overall improvement in peoples’ health. Since availability of scientific evidence does not necessarily mean inherent use in policy or decision-making, deliberate efforts have to be put in place by field epidemiology training programs to increase and track utilization of generated public health evidence. Moving forward, the PHFP needs to come up with a system to consistently identify, evaluate, and track use of generated scientific evidence for evidence-based public health.

## Conclusions

In conclusion, PHFP has ensured timely dissemination of generated public health evidences through various means to a wider range of audience and different levels of health care within Uganda and globally. However, PHFP’s performance in terms of manuscript publications is still limited given the gap between achievements in terms of field products so far executed and publications. The PHFP’s performance could be improved if all clearance guidelines are adhered to and mechanisms to ensure post fellowship publication by the alumni are put in place. Furthermore, field epidemiology training programs, PHFP inclusive need to put in place systems to consistently identify, evaluate, and track use of generated scientific evidence for evidence-based public health.

## Data Availability

The data sets upon which our findings are based belong to the PHFP, are not publically available, and cannot be publically shared.

## Abbreviations

FETP: Field Epidemiology Training Programs
EIS: Epidemic Intelligence Service
PHFP: Public Health Fellowship Program
MoH: Ministry of Health
PEPFAR: U.S. President’s Emergency Plan for AIDS Relief
NRRT: National Rapid Response Team
PHEOC: Public Health Emergency Operations Center
DGHS: Director General of Health Services
NTF: National Task Force
SME: Subject matter expert
NFEC: National Field Epidemiology Conference
US CDC: United States Centers for Disease Control and Prevention
USAID: United States Agency for International Development
CNN: Cable News Network
VoA: Voice of America
NPR: National Public Radio
AFENET: African Field Epidemiology Network
TEPHINET: Training Programs in Epidemiology and Public Health Interventions Network

## References

[1] Overcoming challenges to dissemination and implementation of research findings in under-resourced countries | Reproductive Health | Full Text. https://reproductive-health-journal.biomedcentral.com/articles/10.1186/s12978-018-0538-z. Accessed 23 Apr 2019. 10.1186/s12978-018-0538-z PMC6019998 29945654

[2] Bryce J, Black RE, Walker N, Bhutta ZA, Lawn JE, Steketee RW (2005). Can the world afford to save the lives of 6 million children each year?. The Lancet.

[3] Bridging the “Know-Do” gap meeting on knowledge translation in global health—MEASURE evaluation. https://www.measureevaluation.org/resources/training/capacity-building-resources/high-impact-research-training-curricula/bridging-the-know-do-gap.pdf/view. Accessed 4 Mar 2021.

[4] Brownson RC, Eyler AA, Harris JK, Moore JB, Tabak RG (2018). Getting the word out: new approaches for disseminating public health science. J Public Health Manag Pract.

[5] A case study in serendipity: environmental researchers use of traditional and social media for dissemination. https://journals.plos.org/plosone/article?id=10.1371/journal.pone.0084339. Accessed 23 Oct 2018. 10.1371/journal.pone.0084339 PMC3862833 24349571

[6] Murunga VI, Oronje RN, Bates I, Tagoe N, Pulford J (2020). Review of published evidence on knowledge translation capacity, practice and support among researchers and research institutions in low- and middle-income countries. Health Res Policy Syst.

[7] Ario AR, Wanyenze RK, Opio A, Tusiime P, Kadobera D, Kwesiga B (2018). Strengthening global health security through Africa’s first absolute post-master’s fellowship program in field epidemiology in Uganda. Health Secur.

[8] Ario AR, Makumbi I, Bulage L, Kyazze S, Kayiwa J, Wetaka MM (2019). The logic model for Uganda’s health sector preparedness for public health threats and emergencies. Glob Health Action.

[9] Mboussou F, Ndumbi P, Ngom R, Kamassali Z, Ogundiran O, Van Beek J (2019). Infectious disease outbreaks in the African region: overview of events reported to the World Health Organization in 2018. Epidemiol Infect.

